# Implementation of Research Protocols Assessing Sleep and Circadian Rhythms in Challenging Real-Life Settings: A Critical Appraisal of a Study Protocol, Including Researchers’ Reflections and Participants’ Perspectives

**DOI:** 10.3390/clockssleep8010007

**Published:** 2026-02-09

**Authors:** Carina Fernandes, Ema Leite, Joana E. Coelho, Cátia Reis

**Affiliations:** 1 Centro de Medicina Naval, Portuguese Navy, 2810-001 Almada, Portugal; carina.isabel.fernandes@marinha.pt; 2 NOVA National School of Public Health, Universidade NOVA de Lisboa, 1600-407 Lisbon, Portugal; ema.leite@ulssm.min-saude.pt; 3 Occupational Health Service, ULS Santa Maria, 1649-028 Lisbon, Portugal; 4 Faculdade de Medicina, Universidade de Lisboa, 1649-028 Lisbon, Portugal; 5 Gulbenkian Institute for Molecular Medicine, 1649-028 Lisbon, Portugal; jfecoelho@medicina.ulisboa.pt; 6 Instituto de Saúde Ambiental, Faculdade de Medicina, Universidade de Lisboa, 1649-028 Lisbon, Portugal; 7 Católica Research Centre for Psychological, Family and Social Wellbeing, Faculdade de Ciências Humanas, Universidade Católica Portuguesa, 1649-023 Lisbon, Portugal

**Keywords:** extreme real-life settings, participants’ compliance, research protocols pitfalls, research participants’ satisfaction

## Abstract

Sleep and circadian research in real-life environments is challenging but essential. This study presents the design and implementation of a data-collection protocol in a highly challenging real-life setting over 56 days, examining both researchers’ and participants’ perspectives on its strengths, limitations, and key challenges, and highlighting the lessons learned relevant to future studies in similar contexts. Thirty military submariners completed a questionnaire after the 56-day pre-mission, mission, and post-mission data collection to assess their views on the study and each task. Compliance with measurements (questionnaires, diaries, actigraphy, and blood collections) was quantified and correlated with participants’ answers. Mean global satisfaction was 3.57 ± 0.77 (0–5 scale) and declined across study phases, with a significant change only in the post-mission phase (*p* < 0.001). Higher work satisfaction correlated with better global study satisfaction (ρ = 0.396; *p* = 0.030). Diaries were rated the most burdensome task, while blood collections generated the most polarized responses. Compliance with continuous measurements was high, but these also decreased in the third phase of the study, significantly for actigraphy (*p* < 0.001), although without clear predictors, including satisfaction. In this extreme setting, satisfaction and compliance declined significantly in the final phase of the study, without clear predictive factors. Having different engagement strategies for different work shifts is also an important consideration for future studies.

## 1. Introduction

Circadian rhythms extensively regulate brain and bodily functions, and sleep itself also has an important role in a multitude of physiological processes [1,2]. Disruption of either of these has been robustly linked to negative health outcomes, both in the short and the long term, including increased risk for cardiovascular, metabolic, and mood disorders, cognitive dysfunction, and even cancer [3,4,5,6,7,8,9].

Furthermore, sleep and circadian disruption adversely impact performance, with both individual and societal negative impacts on well-being, productivity, and safety [10,11,12,13,14,15,16,17]. As such, an accurate characterization of these effects and subsequent countermeasures implementation is warranted. This characterization may be achieved from studies in laboratory or real-life settings, each having different pros and cons. Laboratory studies allow for a more complex design, broadness of measurements, and control for confounding factors, with great advantages for biological and physiological modeling and characterization. However, findings from laboratory studies do not translate to real-life settings, and some circumstances are not easily reproducible in a laboratory. Additionally, though confounding factors limit the value of some associations, in real-life settings many factors do interact and influence each other and are important contributors to the ecological validity of the results. It is thus relevant to conduct both types of studies to reinforce complementary validity.

Real-life studies encounter challenges at every phase, from protocol design to participant recruitment, implementation, compliance, and constant adaptation to unexpected factors. This is particularly true when research is conducted in extreme occupational settings and environments. Measurements must conform to available schedules, equipment, and storage possibilities and not interfere with critical activities and safety. Researchers, possible participants, and management/leadership often have very different perspectives on what is feasible or not, due to their different priorities and views. Unforeseen circumstances may and will happen, requiring a flexible approach and prompt adaptations. Confounding factors will be invariably present, and their effect must be acknowledged and mitigated.

On the other hand, the more challenging the real-life research setting is, the more likely it is to imply a sleep and circadian disruption [18], further justifying the need for research in extreme environments.

Despite these challenges, methodological studies in research conducted in real-life settings are scarce. While several studies addressed the conduct of clinical trials and research [19,20,21,22], information on research implementation in occupational/operational settings is much more limited. A few studies evaluating sleep and circadian interventions in occupational settings also assessed implementation enablers and barriers, but most provided limited information [23,24]. Interestingly, a systematic review of such studies found that participant characteristics were the most commonly identified enabler/barrier to implementation [23]. Furthermore, to our knowledge, only one study has previously described a comprehensive data collection protocol implementation in a complex occupational/operational setting, and it did not include participant perspectives [13].

Thus, critical appraisals of research protocols in real-life settings, including researchers’ perspectives, pitfalls, and success factors, are lacking. Also, to our knowledge, participants’ perspectives have not been previously addressed in real-life studies involving healthy subjects.

Therefore, the aims of this study were: (1) to describe a study protocol performed in a challenging real-life setting, encompassing several different measurements; (2) to evaluate participants’ perceptions of the study protocol, both as a whole and regarding each different procedure; and (3) to critically discuss pitfalls and success factors from a lessons-learned perspective.

## 2. Results

### 2.1. Sample General Characteristics

Thirty submariners were included, comprising over 90% of the whole crew, and 100% of eligible crew members.

Subjects were predominantly male (*n* = 27; 90%), with a mean age of 35.6 ± 4.9 years. Most had considerable experience working both in active duty (mean 16.3 ± 5.1 years) and in submarines (mean 6.8 ± 5.6 years), and work satisfaction was reasonably high, with a mean of 6.9 ± 1.6 (1 to 10 scale).

These subjects participated in a 56-day study, divided into three consecutive periods (pre-mission, submarine mission, and post-mission). Discrete questionnaires (4–9 questionnaires/scales, taking 15–45 min, depending on the timepoint) and blood collections were performed once in each study phase, while actigraphy and a sleep-activities diary were collected continuously (daily) throughout the study period. At the end of the study, a final questionnaire evaluated participants’ perceptions of the study and its data collection methods.

Sleep duration, sleep quality, and sleepiness comparisons in pre-mission, mission, and post-mission periods, as well as sample characteristics regarding psychological traits and factors, and their interaction with the sleep variables, have been the subject of a previous report [25].

### 2.2. Participants’ Perspectives

Most participants (*n* = 27; 90%) had participated in a research study before, and the mean global satisfaction with the experience of participation in our study was 3.57 ± 0.77, with points on a rating from 1 “very negative” to 5 “very positive”.

Regarding the total study duration, most (*n* = 19; 63.3%) responded that it was neither too long nor too short, but one third (*n* = 10; 33.3%) rated it as too long, and the maximal duration deemed acceptable for this kind of study was around 30 days (mean 31.8 ± 21.1 days).

**Figure 1:**
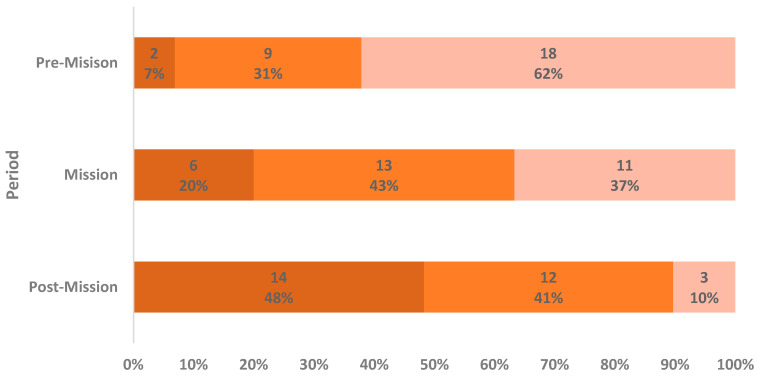
Satisfaction with general data collection across study periods. Ranking of the three study periods from least pleasant/most bothersome (dark orange), to intermediate (orange), to most pleasant/least bothersome (light orange). Labels within the bars represent the number (above) and percentage (below) of participants in each ranking category per study period. There was one missing answer in both pre- and post-mission; valid percentages (rounded to the nearest unit) are reported, excluding the missing answer.

When asked to rank the three study periods from most pleasant/least bothersome to least pleasant/most bothersome for data collection, the pre-mission period was rated by most participants (*n* = 18; 62.1%) as the most pleasant/least bothersome, while the post-mission period was considered (*n* = 14; 48.3%) the least pleasant/most bothersome. As shown in Figure 1, the data suggest a progressive decrease in satisfaction regarding data collection throughout the study period. These differences, calculated with Friedman and Wilcoxon Signed-Rank Tests, were statistically significant and showed a moderate effect size (χ^2^ = 18.348; *p* < 0.001; W = 0.316). Satisfaction did not differ significantly between the pre-mission and mission periods (Z = −1.900; *p* = 0.057). However, satisfaction with data collection was significantly lower in the post-mission phase compared with both pre-mission (Z = −3.706; *p* < 0.001; *r* = −0.688) and mission (Z = −2.459; *p* = 0.014; *r* = −0.457) periods.

When asked specifically to compare the mission to pre- and post-mission periods concerning actigraphy and diary filling, 66.7% (*n* = 20) found the pre- and post-mission periods more bothersome. It is noteworthy that despite the demanding mission environment and schedules, participants did not find this period as challenging as anticipated. Almost unanimously, subjects attributed this to the structured routines during the mission, allowing study tasks to be performed as one more mission task with easy and close access to all materials due to the nature of submarine life and space, as opposed to the pre- and post-mission periods in which these tasks were perceived as more intrusive on normal routines, as materials were cumbersome to carry and therefore easier to forget.

**Figure 2:**
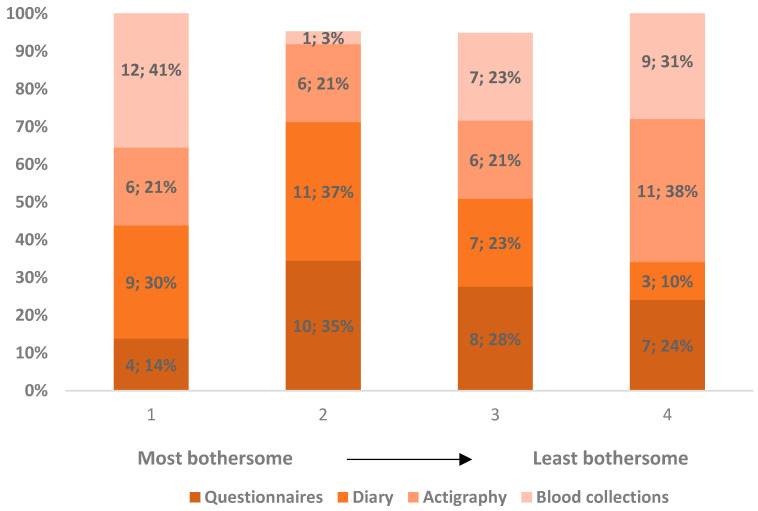
Satisfaction ranking of data collection methods. Ranking of the four data collection methods from 1, most bothersome, to 4, least bothersome. Labels within the bars represent the number and percentage of answers in each rank for each individual method. There was one missing answer; valid percentages (rounded to the nearest unit) are reported, excluding the missing answer. The bars do not correspond exactly to 100% because six subjects attributed the same ranking to two tasks.

Questionnaires as a task were considered fairly neutral (Figure 2) among the study tasks, ranking from the most bothersome to the least; actigraphy was scored as the least bothersome by most; diary filling was the least popular; and, interestingly, blood collections showed the most polarized ratings as it was considered the most bothersome by 41.4% (*n* = 12), but the least by 31.0% (*n* = 9), and with few intermediate ratings. The described differences in these rankings were not statistically significant (χ^2^ = 2.825; *p* = 0.419; Friedman Test). Open-ended justifications provided by participants for the most bothersome task are summarized in Table 1.

**Figure 3:**
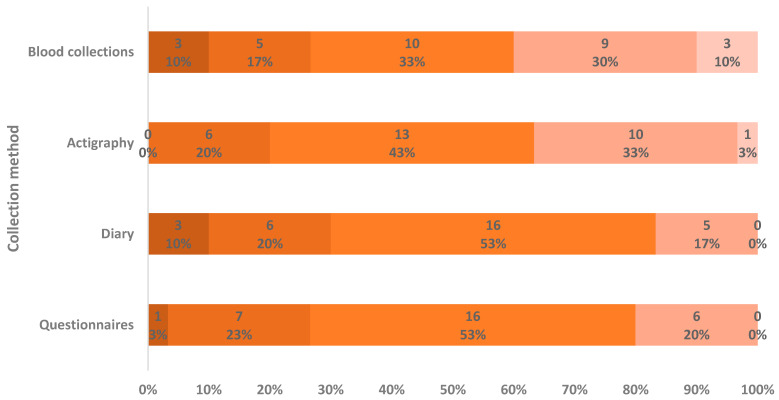
Satisfaction ratings for each data collection method. Rating from 1, very bothersome (darker orange), to 5, very pleasant (lighter orange). Labels within the bars represent the number (above) and percentage (below) of participants who assigned each rating to each task. Percentages were rounded to the nearest unit.

Ratings for each study task are shown in detail in Figure 3. Questionnaires were rated globally as a neutral experience with a mean rating of 2.90 ± 0.76; diary was rated globally as slightly worse with a mean rating of 2.77 ± 0.86; actigraphy was slightly better rated with a mean of 3.20 ± 0.81; and blood collections had a more polarized rating, with a mean of 3.13 ± 1.14, which is in line with the ranking shown in Figure 2. No statistical difference in the distribution of the scores attributed to each measurement (χ^2^ = 4.170; *p* = 0.244; Friedman Test) was apparent. On the other hand, when evaluating the correlation between satisfaction scores for each specific task compared with other tasks, there was a moderate positive correlation only for questionnaires and diary (ρ = 0.536; *p* = 0.002; Spearman’s Rank Correlation Coefficient).

Participants’ reported reasons for disliking study tasks are shown in Table 2.

We wondered if there were concerns or difficulties with continuous measurements specific to the mission period, due to its demanding schedule. Therefore, a closed question asking them to rank different possible reasons in importance was included, and the frequency of answers is described in Table 3 for each task.

The maximum daily time participants considered acceptable for filling out a diary was 5.0 ± 2.92 min. Overall, 73.3% (*n* = 22) reported that a duration of up to 5 min was acceptable, while the maximum number of times per day was on average 1 ± 0.57, while 46.6% (*n* = 14) responded once and no one responded more than twice.

Global and task-specific (Figure 3) satisfaction scores did not associate significantly with age or with work experience, either in military active duty or in the submarines. However, higher work satisfaction was positively correlated with a higher global study satisfaction (ρ = 0.396; *p* = 0.030; Spearman’s Rank Correlation Coefficient).

### 2.3. Compliance with Continuous Measures Data Collection in the Three Study Periods

Compliance with diary and actigraphy collections was reasonably high during pre-mission and mission periods, while this decreased in the post-mission period, as shown in Table 4.

While differences in compliance between periods were not significant for diary, there was a decrease in compliance for actigraphy in the post-mission period compared to both pre-mission (Z = −3.491; *p* < 0.001; *r* = −0.637; Mann–Whitney U Test) and mission periods (Z = −3.878; *p* < 0.001; *r* = −0.708; Mann–Whitney U Test).

Despite no significant difference in compliance for diary filling, the percentage of subjects with complete data (90% or more days with complete data per period) in the pre-mission period was 70% (*n* = 21), 63.3% in the mission period (*n* = 19), and 56.7% in the post-mission period (*n* = 17). This followed the same trend as global satisfaction across the study periods. However, there was no significant correlation between compliance ratio and satisfaction ratings, either for global study satisfaction or task-specific satisfaction (Table 4).

There was also no correlation between compliance ratio and age, work experience (either in active duty or in submarine duty), and work satisfaction. There was also no association between compliance and the perceived length of study duration.

Compliance ratios during the mission period were worse for individuals on shift performing the bulk of night work (Shift 1: 01:00 to 07:00 plus 13:00 to 19:00; *n* = 12) than for those on shift with the longer night rest period (Shift 2: 07:00 to 13:00 plus 19:00 to 01:00; *n* = 13), especially for the diary. Shift 1 had a mean compliance ratio of 0.74 ± 0.31 and only 50% (*n* = 6) of subjects had a complete diary, whereas for Shift 2, the mean compliance ratio was 0.96 ± 0.07 and 84.6% (*n* = 11) of subjects had a complete diary. Although this difference did not reach statistical significance (Z = −1.881; *p* = 0.060; Mann–Whitney U Test), possibly due to the small sample size in each group, we considered it relevant for reflection. There was a similar pattern regarding actigraphy, but with a smaller difference between shifts. Shift 1 had a mean compliance ratio of 0.84 ± 0.35 and Shift 2 had a compliance of 0.93 ± 0.16 (Z = −0.541; *p* = 0.589; Mann–Whitney U Test).

## 3. Discussion

Conducting research in complex real-life settings faces a number of challenges, including participant recruitment and engagement, preventing missing or incomplete data, avoiding technical pitfalls and protocol violations during data collection, and anticipating and/or adapting to challenges and deviations from initial planning. We have conducted a comprehensive research protocol, and participants’ views and compliance regarding this protocol may inform a more successful implementation of future studies.

The existing literature on participant recruitment strategies for clinical research is somewhat limited. Suggested relevant barriers to recruitment include time commitment, reluctance to invasive testing, privacy issues, and a lack of interest. As for enablers, clear communication, supportive staff, flexible study protocols, involvement of family members, monetary gifts and rewards, and perceived direct health benefits are deemed relevant [26,27,28,29]. A synthetic review on interventions for research participant engagement in clinical trials found that useful strategies included building trust between research teams and participants, as well as improving participant comprehension of trial objectives and procedures [30]. However, most interventions did not have a significant effect, creating a challenge in selecting the optimal interventions for increasing research participant engagement, even in clinical research where these have been most studied. Moreover, in the current study, participants are enrolled in an extreme real-life occupational setting, for which there is no prior evidence to support specific recruitment or engagement strategies.

Participant recruitment in our study was very effective, as 100% of eligible subjects participated despite the study’s protocol complexity. The participants were military personnel, and the study was conducted in this context; we must acknowledge a strong bias towards participation by this factor, despite exact recruitment rates not being usually reported in research with active-duty military. Although we did not evaluate participant motivations, some factors can be pointed out for this recruitment success: (1) early and continuous involvement of the leadership (Squadron Command); (2) early involvement of participants, through presentation of the study one month before the inclusion phase, promoting cooperation and group engagement, clear understanding of goals and implications of the study, and providing opportunities for questions and suggestions; (3) having a small and approachable research team in close contact with participants; (4) data collection occurred at the workplace, allowing for flexibility and minimal interference with daily routines; and (5) allocating several days for the initial subject inclusion during the pre-mission phase, which helped establish a personal rapport with participants, adapting to individual schedule constraints and, in turn, allowed troubleshooting and optimization of data collection procedures.

The study duration was circa two months, which a third of participants classified as too long, and, indeed, when specifically asked most responded that the ideal duration would be around 30 days. Accordingly, reported satisfaction decreased progressively in the three study phases, with a significant difference for the post-mission period. Possible disengagement or fatigue with the study was also expressed in a decrease in compliance with continuous measurements in the post-mission phase, especially for actigraphy. Previous studies evaluating adherence to self-monitoring assessments, albeit in different settings, showed that longer-term adherence was higher with lower-burden tools, additional interventionist contact improved consistency, and passively gathered data from wearable devices showed higher long-term engagement [31,32]. This was in contrast with our decrease in actigraphy (wearable device) compliance in the later phase of the study. This later phase of the study (post-mission) was in some respects different from the previous: (1) off-duty and vacation days for some participants; and (2) lack of positive group effect (upon military mission completion) on study-task completion.

Although the influence of group dynamics on several aspects was easily recognizable in the field, the negative effect on task compliance with tasks occurring after the mission period suggested disengagement from the study and from a possible positive group effect on task completion. Indeed, group cohesion has been demonstrated to improve well-being and task performance in military teams, and also in other settings, especially when behavioral aspects of performance were measured; thus, it should be considered in research implementation [33,34].

The use of actigraphy in sleep studies in occupational settings, including the military, aviation, and other transportation systems, has been frequent, although in most cases for much shorter periods and where detailed compliance data was rarely reported. When exclusively considering studies with 30 days or more of continuous actigraphy use, a group of 30 healthy subjects had a 95% compliance on a 112-day evaluation [35], 286 Army cadets wore the actimeter for an average of 70% of days during a 31-day exercise [36], and in a study with submariners undergoing a 30-day mission, 77% had usable data on actigraphy [37]. However, these studies’ designs and reporting on compliance were very heterogeneous and different from the present study.

Compliance with actigraphy may also be negatively affected by certain occupational tasks and technical problems. In our study specifically, some of the devices suffered water damage, either due to recreational (pre- and post-mission) or professional (during mission) activities. Additionally, two subjects (6.7%) developed a skin rash related to the rubber wrist bracelet. Contemplating alternative devices (e.g., chest-worn devices) in the case of specific professional activities and having spare bracelets with different materials (e.g., nylon) for the likelihood of such events (~10% of the subjects) are highly recommended.

Our initial concerns were directed to ascertain that data collection during the mission period was effective and compliance was high, since the research team would be unable to control data collection or even contact the submarine during the mission. To mitigate this, several countermeasures were implemented: (1) a good integration of the protocol tasks in their work assignments; (2) having research allies in the crew, namely, the Executive Officer and the nurse, who frequently reminded participants to fill out the diary and performed basic troubleshooting with the actigraphy devices; and (3) close continuous proximity between participants, enhancing a positive group effect on the behavior of task completion. Our data showed that these measures were effective since there was only a slight, non-significant decline in the quality of data obtained on continuous measurements during the mission period. An interesting finding, although not statistically significant, was the lower diary compliance during the mission among participants whose work periods occurred mainly at night. Although they operated on a 6 h on/6 h off schedule, the submariners attempted to maintain their home-surface clock time. Our results may therefore suggest that participants with a greater degree of circadian misalignment exhibited lower compliance. Several factors may contribute to this pattern, including higher fatigue, less structured routines, and more frequent changes in daily schedules compared with pre- and post-mission periods. We believe this observation warrants consideration in future studies on shift work.

As previously mentioned, the disengagement during the post-period mission was not considered by the research team, and so specific follow-up procedures were not put in place. In the later phase, the group effect was no longer present, while study fatigue continued to accrue. For future studies, mitigation strategies should be implemented that account for the specificities of each phase.

Although we did not find a significant correlation between satisfaction with both the study and specific measurements and compliance, higher work satisfaction was positively correlated with higher global study satisfaction, but not with compliance. The positive correlation could be due to study tasks being somewhat integrated into their daily work activities and mostly occurred at their workplace. The relationship between work satisfaction and satisfaction with research participation in the occupational setting has not been previously assessed. Our data suggests that it would be interesting to further explore this relationship and define possible determinants.

When considering the different study tasks, the diary was the most disliked, mainly because of the daily burden of completing it and the tendency to forget the task; despite this, compliance did not significantly decrease.

Studies evaluating compliance with a digital sleep diary to be filled out twice a day in a 90-day study [38] and a 16-week study [35] showed compliance decreased linearly over time, but notably after the first two months, which might suggest that a longer study duration is a factor for decreased compliance.

In our study, we had to use a paper version of the diaries due to submarine safety requirements and budget constraints. One of the participants lost the diary near the end of the study, and this data was irretrievable. The use of a digital diary/app on individual smartphones could be advantageous, allowing for setting reminders, tracking the daily time of data entry, and enabling a straightforward download to a database. There was some evidence that a digital diary resulted in higher compliance and reduced errors [39]. However, in the extreme environment (submarine) conditions, this was not an option due to specific safety requirements. Reducing the amount of data to fill out is important to lessen missing entries; most participants considered that the better daily completion time should not go beyond 5 min for a diary and preferably only once per day.

Lastly, blood collections presented three main sets of challenges: subject-related, processing, and storage. In this study, subjects’ perceptions of blood collections were the most polarized, having either a very good or very bad rating. Presumably, the good ratings were due to this type of collection being expedited and requiring minimal effort.

Subject-related issues were associated with the individuals’ reactions to blood drawing and, in some cases, difficulty in achieving a good venous puncture that allowed for enough blood collection. The need for blood collection in fasting conditions of several hours, in this case, was necessary for metabolomics analysis, and was generally not appreciated by participants. It was so crucial that individual adherence, sample collection, and immediate processing were maintained for the best possible quality that an improvised wet lab was established at the naval base (work setting), and hence did not require any significant change in their daily routines.

Our study has some limitations. One was that the questionnaire used to assess participants’ perceptions was designed by the research team and was not a validated instrument. However, while the use of a validated instrument was considered, most questions of such questionnaires were directed to clinical contexts [40,41,42] and did not apply to our extreme environment setting.

Furthermore, our goal was to collect the most detailed information possible, specifically on the study and each task performed/requested, which motivated us to develop a specific set of questions more akin to a structured interview and therefore were not intended to provide a single final score (e.g., satisfaction score) based on a high internal consistency between questions. Calculating internal consistency for the five identical Likert scale questions, participants were asked to score satisfaction with each study task specifically, resulting in Cronbach’s alpha of 0.550 which indicated poor internal consistency.

Most questions were independent and aimed at extracting specific opinions for each protocol aspect, and detailed information was provided to guide the implementation of future studies in a similar challenging setting. Nevertheless, this may inform the development of a more structured instrument in the future, targeting real-life setting studies that can be adapted to specific contexts.

This study population was quite specific and homogenous, but perhaps also less generalizable, which constitutes a limitation. It would be relevant to perform similar evaluations in other (less extreme) real-life contexts.

This study’s strengths reflected, first, the implementation of a complex research protocol in a challenging real-life setting with multiple types of measurements. As such, it can provide insights that may be relevant to a wide range of outcomes. Second, evaluating participants’ perspectives on study implementation and satisfaction was both relevant and largely lacking in the literature, despite recognition that it was crucial for promoting satisfaction and successful implementation, particularly in long, continuous monitoring studies. Finally, conducting research in real-life environments rather than controlled laboratory settings enhanced ecological validity, and it allowed for more accurate assessment of human behavior and physiological responses under naturally occurring conditions.

## 4. Materials and Methods

In this section, the whole research protocol that was conducted and constitutes the object of its participants’ perspectives will be described in detail, but the data analysis and results sections will only address data relevant for the aims of the current manuscript, namely those pertaining to perspectives on the protocol itself.

### 4.1. Study Design and Participants

This protocol was used in an observational study aimed at a broad characterization of sleep and circadian disruption in an extreme environment—a military submarine—and the identification of factors involved in observed disruptions. Individual subjects were assessed at different timepoints: a baseline, in normal daily activities, a submarine mission, and the subsequent post-mission recovery. Due to the nature of the diesel-electric submarine used in the mission which are characterized by having small crews, we strived to maximize recruitment success. As such, a suitable target mission was jointly identified with the Submarine Squadron Command. This mission was selected because it allowed for a robust baseline period since there were no operational deployments in the previous months, and, upon return from the mission, no additional deployments for at least one month, permitting an adequate evaluation of the post-mission recovery. Our strategy to maximize compliance was based on a careful presentation of the study objectives, timeline, and tasks required, as well as the research team’s early and close involvement with the participants and ample time for Q&A with the submarine crew involved in the selected mission.

Accordingly, the study was presented in detail by one of the members of the research team (C.F.) to the submarine crew involved in the selected mission. This took place one month before inclusion and data collection initiation and contemplated a detailed description of the study with ample time for questions and clarifications. A second study presentation session was conducted by the research team (C.F., J.C., and the principal investigator, C.R.) on the first day of participants’ inclusion, allowing for complete clarification on possible questions and concerns.

Exclusion criteria were (a) unavailability in any part of data collection, (b) having previously been diagnosed with a sleep disorder, or (c) taking chronic medication that affects sleep or wakefulness. The study was approved by the Ethics Committee of the NOVA National Public Health School (CE-ENSP nº12/2022), and all participants provided informed consent before data collection.

### 4.2. Procedures and Data Collection

Data was collected in real-life conditions during three consecutive periods: pre-mission (P1-baseline), during the mission (P2), and after the mission (P3-recovery) (~56 days total). As described below, there were discrete repeated assessments, performed by the research team in each of the periods, and continuous assessments, performed autonomously by the participants during the whole study duration.

Pre-mission period assessments varied from one to two weeks before the mission started. This allowed for the inclusion of participants on three different days in this period to accommodate individual/professional scheduling preferences and needs. For their convenience, sample collections also occurred at their workplace in the submarine station clinic facilities.

During the pre-mission period, subjects performed their duties on the naval base, having in general a “9-to-5” daytime schedule. The mission consisted of a three-week operational deployment during which participants were continuously aboard the submarine, working on a schedule of 6 h “on” (work itself) and 6 h “off” (rest, meals, leisure, other activities), resulting in two 6 h work/rest periods every 24 h. Work periods were organized in two “mirror” shifts: crew members were either working on shift (1) from 01:00 to 07:00 and from 13:00 to 19:00 or shift (2) from 07:00 to 13:00 and from 19:00 to 01:00. Each submariner kept the same shift throughout the whole mission. A few submariners with specific duties (commanding officer, mechanical engineer, kitchen staff) did not work in shifts, as they instead had extended work hours or on-call duties.

The post-mission recovery period consisted of three weeks after the mission, during which individuals worked in a schedule similar to baseline.

Collected data included repeated measurements, namely questionnaires and blood collections, at three timepoints: at inclusion; immediately upon the return from the mission (at docking); and after the recovery period. In addition, continuous measurements, specifically the sleep and activities diary and actigraphy, were collected during the whole study period (approximately two months). For logistical constraints, during the recovery period, blood collections were performed at two weeks post-mission, while diary and actigraphy collections continued until three weeks post-mission.

**Figure 4:**
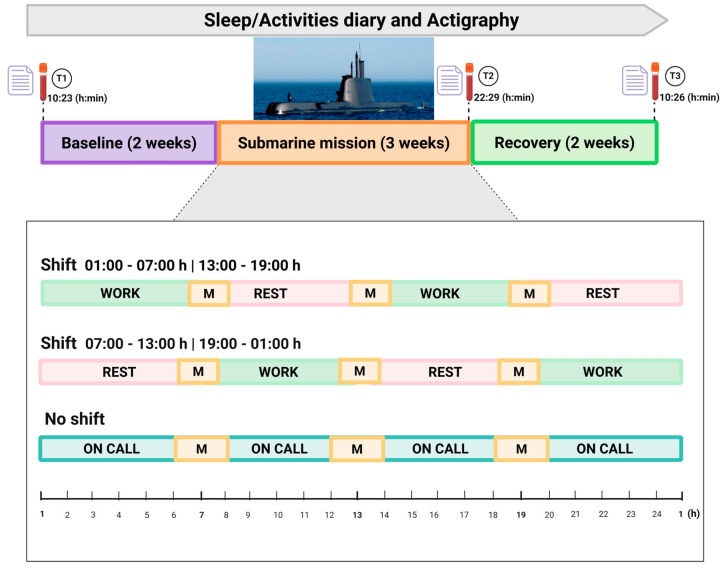
Study diagram with blood collection and questionnaire timepoints. Shift schedules were organized on a 6 h on/6 h off basis: from 07:00–13:00 h to 19:00–01:00 h, and from 13:00–19:00 h to 01:00–07:00 h. Rest periods are in pink. A few participants had extended/on-call working hours. “M” represents mealtime periods for each shift. All blood collections were performed with a minimum of 8 h fasting and processed within 30 min of collection at the naval base. T1, T2, and T3 indicate the clock time of blood sample collection. T2 samples were collected straightaway upon returning from the submarine mission; questionnaires were performed on each blood collection day.

Figure 4 represents the study diagram including measurements and respective timepoints.

### 4.3. Measurements

#### 4.3.1. Questionnaires

Questionnaires were applied at three timepoints and coincided with blood collections. This ensured that there was always at least one member of the research team available for questions, clarifications, and immediate verification of missing data when participants handed in their questionnaires. The questionnaire had 5 to 11 pages, and it took subjects approximately 15 to 45 min to fill out, depending on the timepoint of application.

A more detailed description of the questionnaires used can be found elsewhere [25].

##### Questionnaires Applied Only at Inclusion

▪ General questionnaire to collect demographics, work-related data, and habits: individual characteristics, alcohol, tobacco, and caffeine use, potential exclusion criteria, Navy and submarine service length, and work satisfaction (rated on a scale from 1 to 10, with 10 representing the highest level of satisfaction).
▪ Chronotype: Munich Chronotype Questionnaire [43,44].
▪ Personality traits: Big Five Inventory-10 (BFI-10) [45,46].
▪ Coping mechanisms: Brief-COPE questionnaire [47,48,49].
▪ Type of locus of control: Rotter’s Locus of Control Scale [50,51].

##### Questionnaires Applied Repeatedly at Three Timepoints

▪ Global sleep quality: Jenkins Sleep Scale [52].
▪ Sleepiness: Epworth Sleepiness Scale [53,54].
▪ Fatigue: Fatigue Severity Scale [55,56].
▪ Anxiety and depressive symptoms: Hospital Anxiety and Depression Scale [57,58].

##### Questionnaire Applied Only at the End of the Study

A questionnaire designed by the research team pertaining to the participants’ perceptions regarding the study measurements. It consisted of 21 questions, mostly single-choice closed-ended questions using a Likert scale, or stratification of options according to a magnitude of importance, as well as a few open questions. Questions assessed the perception of the study both globally and specifically for each measurement.

Regarding global study perception:

▪ Previous experience in research studies (yes/no);
▪ Global satisfaction with the study (rating from 1 = “very negative” to 5 = “very positive”);
▪ Ranking study periods (pre-mission, mission, post-mission) from most pleasant/least bothersome to least pleasant/most bothersome for data collection;
▪ Ranking study measurements/tasks (questionnaires, diary, actigraphy, and blood collections) from least bothersome to most bothersome (closed question) and why (open question);
▪ Classifying study duration as “too long”, “not too long nor too short”, “too short” (closed question), and indicating which would be the maximal acceptable duration for this kind of study (open question).

For each specific measurement (questionnaires, diary, actigraphy, and blood sampling):

▪ Rating the experience from 1 = “very unpleasant” to 5 = “very pleasant”;
▪ Indicating unpleasantness factors by both selecting all the applicable from a provided list (closed question) and describing others (open question) if applicable;
▪ Indicating if diary and actigraphy were more bothersome during the mission, during the base periods, or both (closed question) and why (open question);
▪ For the sleep and activities diary, indicating maximal acceptable filling out time (minutes) per day (open question) and maximal acceptable number of times to fill out per day (open question);
▪ Regarding biological samples, indicating if blood collection were preferred, if collection of other biological products like saliva or urine would be preferred, or if it was indifferent;
▪ Specifically, regarding the diary during the mission, ranking the presented reasons for possible discomfort/disturbance in order of decreasing importance (closed question);
▪ Specifically regarding actigraphy during the mission, ranking the presented reasons for possible discomfort/disturbance in order of decreasing importance (closed question).

#### 4.3.2. Sleep and Activities Diary

Participants completed sleep and activities diaries throughout the entire study period. This consisted of a paper diary due to both budget and safety constraints (electronic device use on a military submarine must comply with a set of rules and limitations that made it infeasible in this study). Submariners reported daily sleep schedules, sleep quality, work hours, mealtimes, and caffeine consumption. For every sleep period, they reported bedtime, time to fall asleep, wake-up time, and subjective sleep quality upon awakening (on a scale from 0 to 3, in which 0 was “very good” and 3 was “very bad”). For every work period/shift, participants rated the level of alertness at its beginning and at the end using the Karolinska Sleepiness Scale (KSS), a numerical scale related to subjective drowsiness and rated from 1 = “very alert” to 9 = “very sleepy, great effort to keep awake, fighting sleep” [59]. For caffeine consumption, participants reported the total number of caffeine beverages consumed each day and the timing of consumption.

Upon inclusion, each participant received detailed instructions regarding diary filling. We asked the submarine’s Executive Officer and the nurse to remind the crew daily to fill out the diary. This also promoted a group effect on adherence.

#### 4.3.3. Actigraphy

Participants wore an actimeter (ActTrust^®^, Condor Instruments^TM^, São Paulo, Brazil) on their non-dominant wrist for the entire study period (approximately 56 days). This device continuously collected activity (Proportional Integral Mode—PIM), environmental light, and temperature at a sampling rate of 60 s. Off-wrist periods were identified using ActStudio^®^ software (version 2.2.2) through visual inspection. Days with more than 4 h of off-wrist periods were excluded from the analyses [60].

Participants were carefully instructed to wear the actimeter continuously during the whole study period and to remove it only for activities involving water. All devices were fully charged when given to each participant. However, to prevent events of battery insufficiency, especially during the mission, two device chargers were provided along with instructions on how to charge the device to the submarine’s Executive Officer.

#### 4.3.4. Blood Samples Collection

Blood samples were collected at three specific timepoints, with one single sample of venous blood drawn from each subject at each timepoint: at inclusion, immediately after submarine docking upon returning from the mission, and at two weeks post-mission.

The blood samples were processed to obtain two different constituents: Peripheral Blood Mononuclear Cells (PBMCs) for transcriptomics of clock genes, and serum for metabolomics. The procedures were adapted from the SOPs provided by Professor Achim Kramer, Laboratory of Chronobiology, Charité Universitatsmedizin, Berlin, Germany, and by Professor Debra Skene, Centre for Chronobiology, School of Biomedical and Life Science, University of Surrey, UK, respectively. Following the exact instructions from the laboratories that would perform the analyses guaranteed higher sample quality. However, this required one qualified researcher (J.C.) handling each blood sample in two separate processing protocols.

Due to the need for immediate processing, an ad hoc collection point and improvised lab were established in the submarine station clinic facilities at the naval base, with the necessary equipment being transported to and placed in loco, including three centrifuges (having access to several centrifuges was crucial, since part of the sample processing required two-step centrifugations), general laboratory materials, and dry ice containers. This allowed for collections to happen with minimal disruption of the submariners’ routines at all timepoints, particularly for immediate collection upon exiting the submarine when docking at the naval base at the end of the mission. It also allowed for immediate sample processing and preservation in dry ice for subsequent storage at −80 °C.

To ensure a swift and effective process, the space was previously organized and a workflow was planned. Two nurses performed blood collections, one researcher confirmed identification codes and registered clock times for collection, centrifugation, and temporary storage, and one experienced laboratory scientist performed all the sample processing. Timings of blood collection, centrifugation start, and sample freezing were noted down to register any deviations from the maximal timing of one hour for complete processing of blood to freeze. After collection and sample processing were concluded, samples were kept in a dry ice box and promptly stored at −80 °C.

Since blood collection had to be performed while fasting, the research team always provided snacks/food and coffee for all individuals after completion of the task.

### 4.4. Data Analysis

Data concerning the questionnaire of participants’ perceptions was analyzed through descriptive statistics and frequencies, means, and standard deviations were presented. Due to most variables not following a normal distribution (assessed using the Shapiro–Wilk Test), non-parametric tests were subsequently used.

Satisfaction scores with different study periods and measurements were compared with the Friedman Test and Wilcoxon Signed-Rank Test (effect sizes calculated with Kendall’s W and *r* = Z/√(n), respectively).

Associations between global and task-specific satisfaction scores, as well as those with demographic variables were evaluated with Spearman’s Rank Correlation Coefficient.

Compliance with continuous measures was defined for each participant as an average of their daily compliance in each period. Regarding the diary, for each day, a score of 0 (no data), 0.5 (partial data), or 1 (complete data) was assigned, and the average score for each period was calculated. Regarding actigraphy, for each day, a score of 0 (invalid data, with more than 4 h of off-wrist periods) or 1 (valid data) was assigned, and the average score for each period was calculated. Compliance data for diary was also subsequently computed as a categorical variable, with the following categories: 0 = no compliance (0.10 or less average score for the period), 1 = partial compliance (between 0.11 and 0.89 average score for the period), and 2 = total compliance (equal or higher than 0.90 average score for the period).

Significant differences in compliance with diary and actigraphy in the three periods were assessed through the Friedman Test and Wilcoxon Signed-Rank Test (effect sizes calculated with Kendall’s W and *r* = Z/√(n), respectively).

Associations between compliance with continuous measurements (diary and actigraphy), satisfaction ratings attributed to the study and its tasks, and demographic variables were evaluated using Spearman’s Rank Correlation Coefficient or the Mann–Whitney U Test (effect size calculated with *r* = Z/√(n) for the latter), as appropriate.

All analyses were performed using IBM SPSS version 29, and statistical significance was set at 0.05 for all calculations.

## Figures and Tables

**Table 1:** Participants’ reasons for selecting each study task as the most bothersome.

Task	Justifications
Questionnaires	*“Too long”*
Diary	*“Having to carry the diary around all the time.”*
	*“Paper format.”*
	*“Having the concern of always checking the time of the activities.”*
	*“The care needed for precision of the information registered and concern about possible errors.”*
	*“Prone to forgetfulness.”*
	*“Having to fill out the diary every day is cumbersome due to the duration of the study.”*
	*“Although easy to fill out, with time it becomes boring and easy to forget.”*
	*“Having to dedicate time every day to filling out the diary.”*
Actigraphy	*“Causes discomfort in some work activities.”*
	*“I don’t like to always wear a watch.”*
	*“It is uncomfortable to wear the device during sleep.”*
	*“Having to worry about remembering to always put it on again after removal.”*
	*“Could not wear my watch at the same time.”*
	*“Having to avoid device contact with water.”*
Blood collections	*“The need for prolonged fasting periods.”*
	*“I don’t like to have blood draws.”*
	*“I don’t like needles.”*
	*“Too many blood draws.”*
	*“I get dizzy and feel malaise when having blood draws.”*
	*“My veins are difficult to find, and blood draws are always difficult.”*

**Table 2:** Participants’ reported reasons for disliking each study task.

Task	Reason	Frequency (*n*, %)
Questionnaires	Nothing in particular	8 (26.7)
	Being long	10 (33.3)
	Having to fill out questionnaires several times during the study period	12 (40.0)
	Not understanding the purpose of these questionnaires quite well	1 (3.3)
Diary	Nothing in particular	7(23.3)
	Taking too long to fill out	3 (10.0)
	Having to fill out the diary every day	17 (56.7)
	Being in paper format, I would prefer a digital version	9 (30.0)
Actigraphy	Nothing in particular	6 (20.0)
	The worry of having to wear the device all the time	8 (26.7)
	The use was uncomfortable	10 (33.3)
	Not being able to wear a watch/smartwatch at the same time	9 (30.0)
Blood collections	Nothing in particular	19 (63.3)
	Being painful	0 (0.0)
	Disliking blood draws	4 (13.3)
	Involving repeated samples/draws	6 (20.0)

For each collection method, participants were instructed to select all the applicable factors; more than one response per participant was therefore possible. The number and percentage of participants who selected each response were presented.

**Table 3:** Participants’ ranked reasons for disliking continuous data collection measures, specifically during the mission period.

Task	Reason	Frequency (*n*, %)
Diary	Having daily concern about filling it out	17 (56.7)
	Difficulty remembering to fill out the diary	4 6 (20.0)
	Worry about losing or damaging the diary	4 (13.3)
	Preference for a digital diary	3 (10.0)
	The information required is too detailed	1 (3.3)
	Taking a lot of time to fill out	1 (3.3)
Actigraphy	Worrying about not damaging the device	15 (50.0)
	Not being able to wear a watch/smartwatch at the same time	6 (20.0)
	Discomfort in daily tasks performance	3 (10.0)
	General discomfort, irrespective of task	3 (10.0)
	Worrying about not removing the device, or always remembering to put it back after removal	2 (6.7)
	Worrying about keeping the device uncovered at all times	1 (3.3)

Reasons were ranked from most to least important. The frequency and number of participants selecting each of the reasons as the most important are presented. The total number of responses does not correspond exactly to the number of participants (*n* = 30) because four participants ranked two reasons as the most important.

**Table 4:** Compliance with continuous measurements in each study period.

	Diary			Actigraphy		
	Pre-Mission	Mission	Post-Mission	Pre-Mission	Mission	Post-Mission
Compliance ratio (mean ± SD)	0.87 ± 0.24	0.83 ± 0.26	0.78 ± 0.33	0.84 ± 0.28	0.85 ± 0.31	0.57 ± 0.39
	χ^2^ = 1.949; *p* = 0.377; W = 0.032			χ^2^ = 22.415; *p* < 0.001; W = 0.374		
Compliance vs satisfaction	ρ = 0.294 *p* = 0.115	ρ = −0.010 *p* = 0.959	ρ = −0.039 *p* = 0.839	ρ = −0.047 *p* = 0.805	ρ = −0.046 *p* = 0.809	ρ = −0.045 *p* = 0.813

Comparison of compliance ratio between periods was assessed with the Friedman Test, and the relationship between compliance ratio and satisfaction scores attributed to diary and actigraphy was evaluated using Spearman’s Correlation Coefficient (statistical significance was set at *p* < 0.05).

## Data Availability

The dataset presented in this article is not readily available due to the sensitive nature of this study population. Requests to access the dataset should be directed to the corresponding author.
